# Inflammatory and Neuropathic Nociception is Preserved in GPR55 Knockout Mice

**DOI:** 10.1038/s41598-017-01062-2

**Published:** 2017-04-20

**Authors:** Lawrence M. Carey, Tannia Gutierrez, Liting Deng, Wan-Hung Lee, Ken Mackie, Andrea G. Hohmann

**Affiliations:** 1 Department of Psychological and Brain Sciences, Indiana University, Bloomington, IN USA; 2 Program in Neuroscience, Indiana University, Bloomington, IN USA; 3 Interdisciplinary Biochemistry Program, Molecular and Cellular Biochemistry Department, Indiana University, Bloomington, IN USA; 4 Gill Center for Biomolecular Science, Indiana University, Bloomington, IN USA

## Abstract

The G-protein coupled receptor GPR55 has been postulated to serve as a novel cannabinoid receptor. A previous report indicated that GPR55 knockout mice fail to develop mechanical hyperalgesia, suggesting a pro-nociceptive role for GPR55 in the control of nociceptive responding. However, GPR55 knockout mice remain incompletely characterized in models of pathological pain. Here we provide a comprehensive assessment of responses of GPR55 knockout and wild-type mice to mechanical and thermal (heat, cold) stimulation in multiple, mechanistically distinct models of inflammatory and neuropathic pain. Inflammatory sensitization was produced by intraplantar administration of capsaicin, formalin or complete Freund’s adjuvant. No differences in responding were detected between GPR55 knockout and wild-type mice in any model of inflammatory nociception assessed. Neuropathic pain was induced by partial sciatic nerve ligation (which induces hypersensitivity to mechanical, cold and heat stimulation) or by treatment with the chemotherapeutic agent paclitaxel (which induces hypersensitivity to mechanical and cold stimulation only). No differences were observed between GPR55 knockout and wild type mice in either development or maintenance of neuropathic nociception in either neuropathic pain model. In conclusion, genetic deletion of GPR55 did not alter the development of pathological pain in adult mice in any chronic pain model evaluated.

## Introduction

The orphan G-protein coupled receptor 55 (GPR55) has been postulated to serve as a novel cannabinoid receptor^1–3^ and possible therapeutic target in a wide array of pathophysiological conditions. For example, GPR55 has been implicated in cancer cell proliferation and disease progression^4^, bone growth and osteoclast function^5^, metabolism^6, 7^, and nociception^8^. However, elucidating the physiological importance of the GPR55 receptor has remained elusive as a wide range of structurally distinct compounds including endogenous lipid mediators, phytocannabinoids and synthetic cannabinoid ligands exert pharmacological effects at GPR55. The problem posed by the lack of selective ligands for GPR55 (i.e. virtually all of which are known to exert pharmacological effects at other targets) is compounded by inconsistencies in the literature regarding the pharmacological effects that ligands exert through GPR55^9^.

GPR55 is expressed broadly, though at quite low levels throughout the CNS^3, 10^, and in large diameter dorsal root ganglion neurons^11^, where GPR55 agonists promote increases in Ca^+2^ release from intracellular stores. The distribution of GPR55 and the potential for enhancement of neuronal excitability suggest that GPR55 may play a role in nociceptive processes. As the lack of selective ligands for GPR55 precludes the use of pharmacological tools to probe its function, the role of GPR55 in the development of pathological pain states was investigated in mice with the gene encoding the GPR55 receptor deleted (GPR55 KO)^8^. Interestingly, these mice failed to develop mechanical hyperalgesia following either partial sciatic nerve ligation (PSNL) or inflammation induced by intraplantar (i.pl.) administration of Freund’s complete adjuvant (CFA), whereas robust mechanical hypersensitivity developed in corresponding wild-type (WT) littermates that received the same nociceptive challenge. The lack of mechanical hypersensitivity reported in GPR55 KO mice was accompanied by limited alterations in cytokine profiles induced by CFA that did not correlate with the development of hyperalgesia.

To our knowledge, the literature employing GPR55 KO mice to investigate a role for GPR55 in nociception and pathological pain is limited to a single published report employing a single dependent measure to assess nociception (i.e. Randall-Selitto test of mechanical hypersensitivity). Nonetheless, based largely upon this report, GPR55 is postulated to be inherently nociceptive because GPR55 KO mice display an analgesic phenotype (i.e. fail to develop mechanical hypersensitivity to inflammatory and neuropathic pain)^8^. For this reason, the present study sought to further characterize the profile of nociceptive behaviors in GPR55 KO mice by measuring responsiveness of these mice to multiple modalities of cutaneous stimulation (mechanical, cold and heat) in a battery of mechanistically distinct inflammatory (induced by formalin, capsaicin and CFA) and neuropathic (induced by traumatic nerve injury and chemotherapy treatment) pain states. Capsaicin, the pungent ingredient in hot chili peppers, is a transient receptor potential cation channel subfamily V member 1 (TRPV1) agonist and produces nocicfensive behavior and heat hypersensitivity, both of which are associated with peripheral sensitization, as well as mechanical hypersensitivity which is associated with central sensitization and secondary hyperalgesia^12, 13^. Formalin induces a biphasic pattern of pain response which involves primary afferent activation (i.e. primarily in phase 1) as well as NMDAR-mediated central nervous system sensitization (i.e. primarily in phase 2)^14–16^. CFA induces a long duration unilateral peripheral edema as well as hypersensitivity to mechanical and heat stimulation that is associated with both peripheral and central sensitization, involving activation of multiple cell types, signaling cascades and both NMDAR-dependent and NMDAR-independent mechanisms^17–19^. In separate studies, we evaluated GPR55 KO and WT mice for responsiveness to mechanical and thermal (i.e. heat) stimulation in models of inflammatory pain induced by intraplantar injection of capsaicin or CFA. We evaluated nocifensive behavior (i.e. time spent licking, lifting and flinching the injected paw) induced by intraplantar injection of either capsaicin or formalin. We also evaluated GPR55 KO and WT mice in mechanistically distinct models of neuropathic pain including a model of chemotherapy-induced neuropathic pain induced by paclitaxel treatment and a model of traumatic nerve injury induced by PSNL^8^. PSNL produces a neuropathic pain state marked by hypersensitivity to mechanical, cold and heat stimulation^20^ whereas toxic challenge with the chemotherapeutic agent paclitaxel produces hypersensitivity to mechanical and cold stimulation only^21^. Overall, our findings suggest that genetic deletion of GPR55 does not markedly alter the development or maintenance of inflammatory or neuropathic pain in adult mice tested in mechanistically distinct models of pathological pain with a wide array of cutaneous (mechanical, cold, heat) stimuli.

## Results

### Male GPR55 KO and WT mice display decreases in rotarod performance

**Figure 1:**
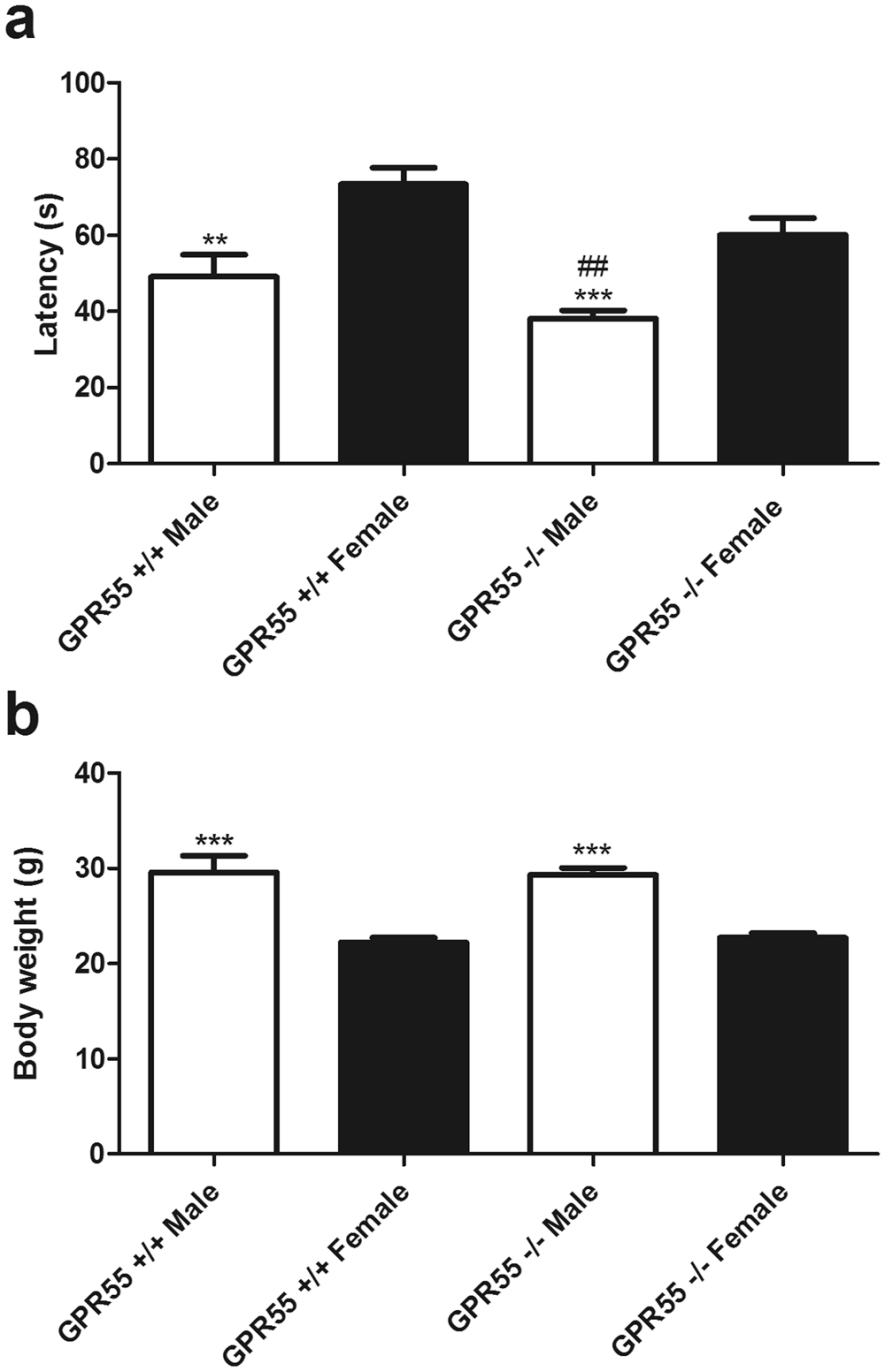
Male mice show decreases in rotarod performance. Male GPR55 KO and WT mice display decreased latencies to descent relative to female GPR55 KO and WT mice, (**a**). Data are expressed as mean ± SEM (n = 9–15 per group). Two-way ANOVA, 2-tailed t-test with Welch’s correction. ***p < 0.001, **p < 0.01vs. female WT, ^##^p < 0.01 vs. female GPR55 KO. Male mice display higher body weights relative to female mice irrespective of genotype (**b**). Data are expressed as mean ± SEM (n = 9–15 per group). Two-way ANOVA, two-tailed *t*-test.

To assess rotarod performance, the longest latency to descent for each mouse was obtained and averaged for each group to conform to the dependent measure used by Staton and colleagues^8^ in their statistical analyses. Sex (F_1,43_ = 26.62, p < 0.0001; Fig. 1a) and genotype significantly affected motor performance (F_1,43_ = 7.388, p < 0.01; Fig. 1a) and the effect of interaction approached significance (p = 0.06). Post-hoc analyses revealed that, relative to female WT mice, male GPR55 KO (p < 0.001), and male WT mice (p < 0.01) displayed shorter latencies to descend from the rotarod. Male GPR55 KO mice also displayed shorter descent latencies than female GPR55 KO mice (p < 0.01).

Body weight also differed in male and female mice (F_1,43_ = 69.15, p < 0.0001; Fig. 1b), but there was no effect of genotype (p > 0.8) or significant interaction between sex and genotype (p > 0.6). Post-hoc analyses revealed that male GPR55 KO and WT mice weighed more than female GPR55 KO and WT mice (p < 0.001).

### Female GPR55 KO display decreased withdrawal latencies to hot plate stimulation

**Figure 2:**
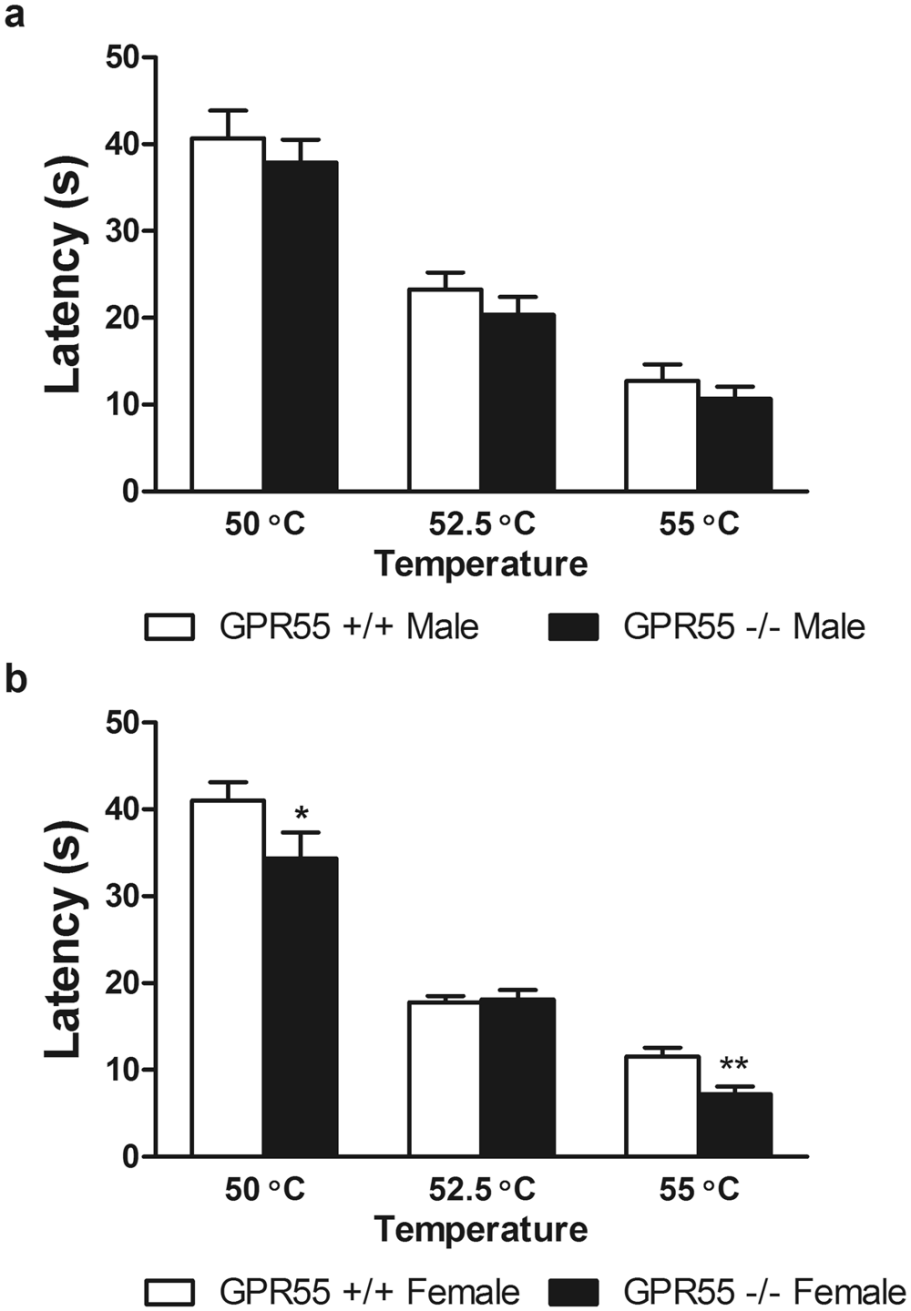
Female, but not male, GPR55 KO mice display decreased thermal paw withdrawal latencies to hot plate stimulation. Female GPR55 KO mice displayed decreased withdrawal latencies to the onset of nociceptive behavior in the hot plate assay at 50 °C and 55 °C relative to female WT mice. Data are expressed as mean ± SEM (n = 9–15 per group). Two-way ANOVA, one-tailed t-test. **p < 0.01, *p < 0.05 vs. female WT.

In male mice, thermal response latencies were altered by hot plate temperature (F_2,34_ = 107.8, p < 0.0001; Fig. 2a), but there was no effect of genotype (p > 0.2; Fig. 2a) or interaction (p > 0.9; Fig. 2a). In female mice, withdrawal latencies were impacted by hot plate temperature (F_2,52_ = 144.9, p < 0.0001; Fig. 2b) and genotype (F_1,52_ = 7.736, p < 0.01; Fig. 2b), but no significant interactions were detected (p > 0.1; Fig. 2b). As previous reports indicated that female GPR55 KO mice may show lower baseline nociceptive latencies to heat stimulation compared to their WT counterparts in the hot plate test, we conducted planned comparison t-tests to further evaluate the presence of genotype differences in female mice at each hotplate temperature. Female GPR55 KO mice displayed decreased withdrawal latencies at 50 °C (t_26_ = 1.847, p < 0.05; Fig. 2b) and 55 °C (t_26 = _3.158, p < 0.01; Fig. 2b) relative to WT female mice but not at 52.5 °C.

### Capsaicin-evoked nocifensive behavior, heat hyperalgesia and mechanical allodynia do not differ between GPR55 KO and WT mice

**Figure 3:**
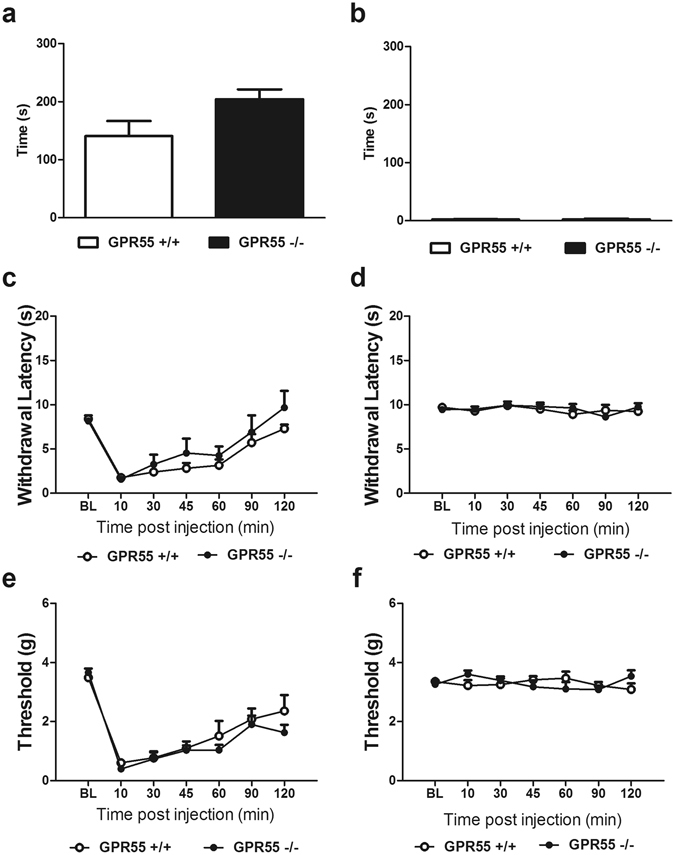
GPR55 KO and WT mice display comparative levels of capsaicin-evoked nocifensive behavior, mechanical and thermal hyperalgesia. (**a**) A trend toward higher levels of capsaicin-evoked nocifensive behavior was observed in GPR55 KO relative to WT mice but (**b**) no difference in nocifensive behavior was observed between genotypes in mice receiving vehicle (i.pl.). Data are expressed as mean ± SEM (n = 6 per group). 2-tailed t-test. Levels of capsaicin-evoked (**c**) thermal and (**e**) mechanical hypersensitivity do not differ between GPR55 KO and WT mice. (**d**) Thermal paw withdrawal latencies and (**f**) mechanical paw withdrawal thresholds did not differ between GPR55 KO and WT mice receiving vehicle (i.pl.). Data are expressed as mean ± SEM (n = 6 per group). 2-way ANOVA.

Levels of nocifensive behavior evoked by intraplantar (i.pl.) capsaicin (p > 0.06; Fig. 3a) Fig. 3b) did not differ reliably between GPR55 KO and WT mice. In fact, a trend toward heightened capsaicin-evoked nocifensive behavior was observed in GPR55 KO vs. WT mice. Similarly, there were no differences in responses between GPR55 KO and WT mice that received i.pl. injections of vehicle in lieu of capsaicin (p > 0.9; Fig. 3b). I.pl. capsaicin decreased thermal paw withdrawal latencies to heat stimulation in all mice (F_6,60 = _15.34, p < 0.0001; Fig. 3c). However, thermal paw withdrawal latencies did not differ between genotypes (p > 0.1; Fig. 3c), and the interaction between capsaicin treatment and genotype was not significant (p > 0.8; Fig. 3c). Moreover, vehicle injection (i.pl.) did not alter thermal paw withdrawal latencies (p > 0.2; Fig. 3d), and there was no significant effect of genotype (p > 0.7; Fig. 3d), or interaction (p > 0.5; Fig. 3d).

I.pl. capsaicin decreased mechanical paw withdrawal thresholds in all mice (F_6,60 = _32.28, p < 0.0001; Fig. 3e). However, genotype did not alter capsaicin-evoked mechanical hypersensitivity (p > 0.3; Fig. 3e), and the interaction was not significant (p > 0.6; Fig. 3e). I.pl. vehicle also failed to alter mechanical paw withdrawal thresholds (p > 0.8; Fig. 3f), and there was no effect of genotype (p > 0.8; Fig. 3f) or interaction (p > 0.09; Fig. 3f).

### Formalin-evoked pain behavior does not differ in WT and GPR55 KO mice

**Figure 4:**
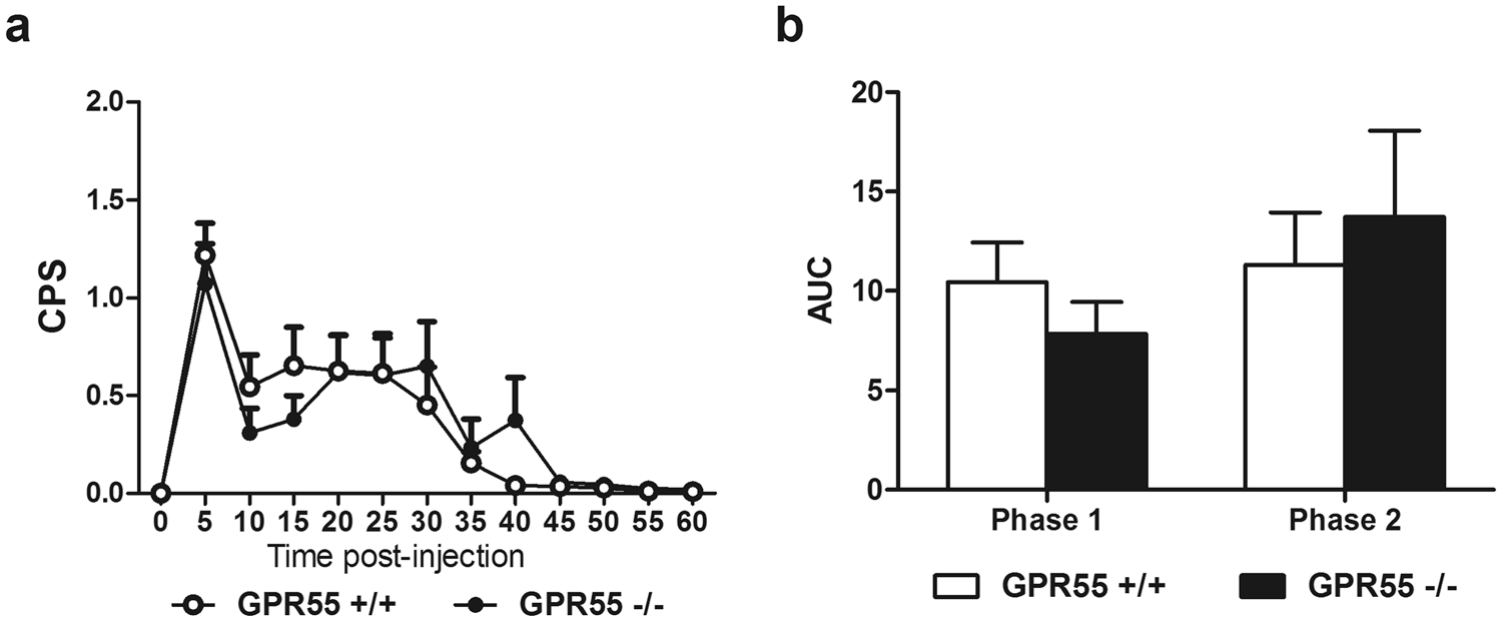
Levels of formalin-evoked nociceptive behaviors do not differ between GPR55 KO and WT mice. GPR55 KO and WT mice display similar levels of pain behavior in response to intraplantar formalin (**a**,**b**). Data are expressed as mean ± SEM (n = 6 per group). 2-way ANOVA. CPS: composite pain score, AUC: area under the curve.

Formalin (i.pl.) increased composite pain scores in both GPR55 KO and WT mice (F_12,60 = _17.14, p < 0.0001; Fig. 4a). However, genotype did not affect composite pain scores (p > 0.9; Fig. 4a) and the interaction was not significant (p > 0.5; Fig. 4a). Neither phase (p > 0.1; Fig. 4b) nor genotype (p > 0.9; Fig. 4b) affected the area under the curve of formalin-evoked pain, and the interaction was not significant (p > 0.2; Fig. 4b).

### Intraplantar CFA elicits equivalent levels of mechanical hypersensitivity and paw edema in GPR 55 KO and WT mice

**Figure 5:**
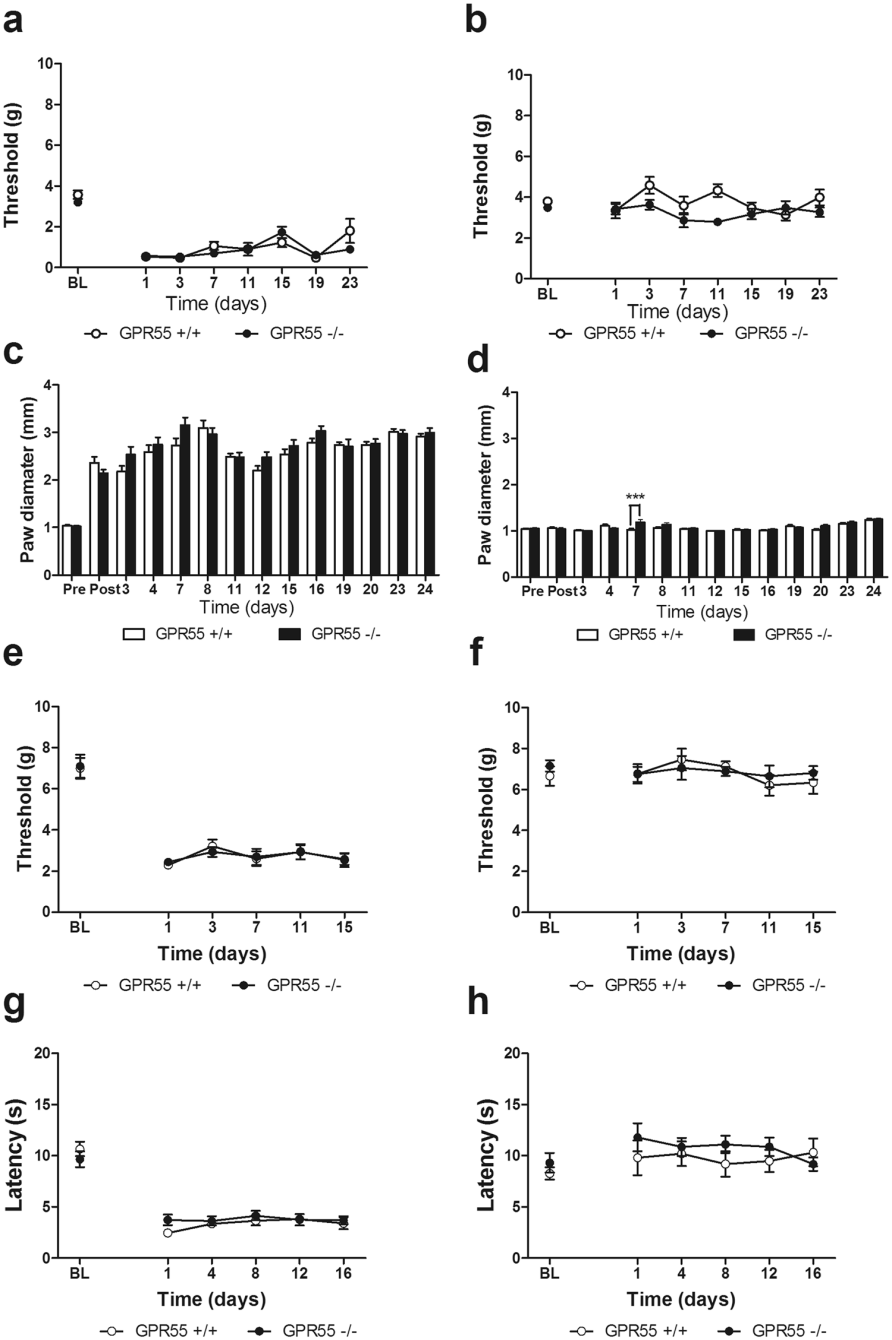
CFA elicits comparable levels of mechanical and thermal hypersensitivity in GPR55 KO and WT mice. Levels of CFA-evoked mechanical hypersensitivity do not differ between GPR55 KO and WT mice (**a**). CFA does not elicit mechanical hypersensitivity in the paw contralateral to CFA administration (**b**). Paw diameters were similar in the CFA-injected (ipsilateral) paw in GPR55 KO and WT mice (**c**). Paw diameters were elevated in GPR55 KO relative to WT mice in the paw contralateral to CFA administration on day 7 only (**d**). Data are expressed as mean ± SEM (n = 8–9 per group). ***p < 0.001 vs. WT, 2-way ANOVA, Bonferroni post-hoc. In an independent replication, GPR55 KO and WT mice develop similar levels of mechanical (**e**), and thermal hypersensitivity (**g**) following i.pl. CFA. Mechanical (**f**), and thermal (**h**) hyperalgesia do not develop in the paw contralateral to CFA administration. Data are expressed as mean ± SEM (n = 8 per group). 2-way ANOVA.

CFA (i.pl.) decreased mechanical paw withdrawal thresholds (F_7,105_ = 38.61, p < 0.0001; Fig. 5a) to similar levels in GPR55 KO and WT mice (p > 0.3; Fig. 5a), and the interaction was not significant (p > 0.07; Fig. 5a). In the paw contralateral to CFA administration, CFA did not alter mechanical withdrawal thresholds (p > 0.09; Fig. 5b). Although genotype-dependent differences in mechanical hypersensitivity were observed in the contralateral paw (F_1,105 = _10.93, p < 0.01; Fig. 5b), the interaction between time and genotype was not significant (p > 0.05; Fig. 5b). Paw diameters were also similar in the CFA-treated paw (F_13,195_ = 46.6, p < 0.0001; Fig. 5c) in GPR55 KO and WT mice (p > 0.1; Fig. 5c), and the interaction was not significant (p > 0.1; Fig. 5c). In the paw contralateral to CFA administration, paw diameter varied through the observation period (F_13,195_ = 15.04, p < 0.0001; Fig. 5d). GPR55 KO and WT mice displayed differing paw diameters (F_1,195_ = 10.81, p < 0.01; Fig. 5d), and this effect was time dependent (F_13,195_ = 2.6, p < 0.01; Fig. 5d). However, subsequent evaluation of the source of the interaction revealed that GPR55 KO mice displayed larger paw diameters relative to WT mice in the paw contralateral to CFA administration on day 7 only (p < 0.001), possibility reflective of transient variability in measurement by the experimenter on this day.

In an independent replication performed in a separate cohort of all male mice by a separate experimenter blinded to genotype, GPR55 KO and WT mice developed mechanical hypersensitivity following CFA (i.pl.) administration (F_5,70_ = 57.51, p < 0.0001; Fig. 5e). Again, GPR55 KO and WT mice displayed similar levels of CFA-induced mechanical hypersensitivity in this separate cohort (p > 0.9; Fig. 5e), and the interaction was not significant (p > 0.9; Fig. 5e). CFA did not alter paw withdrawal thresholds in the paw contralateral to CFA administration (p > 0.4; Fig. 5f), and there was no significant effect of genotype (p > 6; Fig. 5f) or interaction (p > 0.8; Fig. 5f). Paw diameters were not measured in this experiment to eliminate possible handling stress that could potentially impact changes in endogenous analgesic tone.

CFA produced heat hypersensitivity in the inflamed paw (F_5,70_ = 65.74, p < 0.0001; Fig. 5g). However, CFA-induced thermal paw withdrawal latencies to heat did not differ between GPR55 KO and WT mice (p > 0.6; Fig. 5g), and the interaction between genotype and post-injection responsiveness was not significant (p > 0.2; Fig. 5g). In the paw contralateral to CFA administration, thermal paw withdrawal latencies were not altered (p > 0.5; Fig. 5h), and there was no effect of genotype (p > 0.09; Fig. 5h) and no significant interaction (p > 0.5; Fig. 5h).

### GPR55 KO and WT mice display similar levels of paclitaxel-induced mechanical and cold allodynia

Because responsiveness of WT and KO mice receiving the cremophor vehicle in lieu of paclitaxel did not differ from each other, these groups were pooled into a single control group for statistical evaluation of the impact of paclitaxel on GPR55 KO and WT mice.

**Figure 6:**
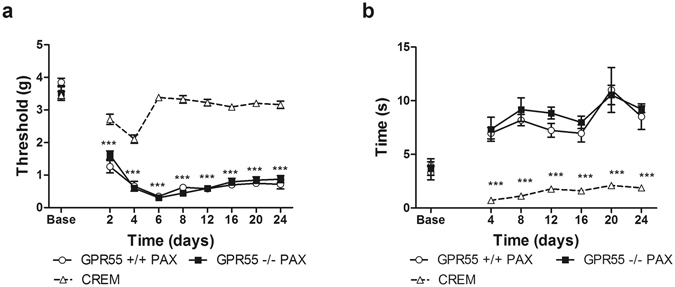
GPR55 KO and WT mice develop similar levels of paclitaxel-induced mechanical and cold allodynia. GPR55 KO and WT mice display similar levels of paclitaxel-induced mechanical (**a**) and cold (**b**) allodynia. Responding did not differ in GPR55 KO or WT mice that received the cremophor vehicle (CREM) in lieu of paclitaxel, and these groups were pooled into a single control (paclitaxel-untreated) group. Data are expressed as mean ± SEM (n = 5–8 per group). ***p < 0.001 GPR55 KO PAX and WT PAX vs. cremophor, 2-way ANOVA, Bonferroni post-hoc.

In both GPR55 KO and WT mice, paclitaxel treatment induced hypersensitivity to mechanical (F_8,88_ = 153.3, p < 0.0001; Fig. 6a) and cold (F_6,66_ = 16.31, p < 0.0001; Fig. 6b) stimulation. However, GPR55 KO and WT mice displayed similar levels of paclitaxel-induced mechanical (p > 0.8; Fig. 6a) and cold (p > 0.4; Fig. 6b) allodynia, and the interaction between genotype and test day response was not significant for either mechanical (p > 0.2; Fig. 6a) or cold (p > 0.9; Fig. 6b) responsiveness. No differences in baseline responding to mechanical or cold stimulation were observed between genotypes. Both GPR55 KO and WT mice treated with paclitaxel displayed decreased paw withdrawal thresholds on days 2–24 (p < 0.001; Fig. 6a) and increased response time to cold stimulation on days 4–24 (p < 0.001; Fig. 6b) relative to cremophor-treated mice.

### GPR55 KO and WT mice develop similar levels of hypersensitivity to mechanical, heat and cold stimulation in response to partial sciatic nerve injury

**Figure 7:**
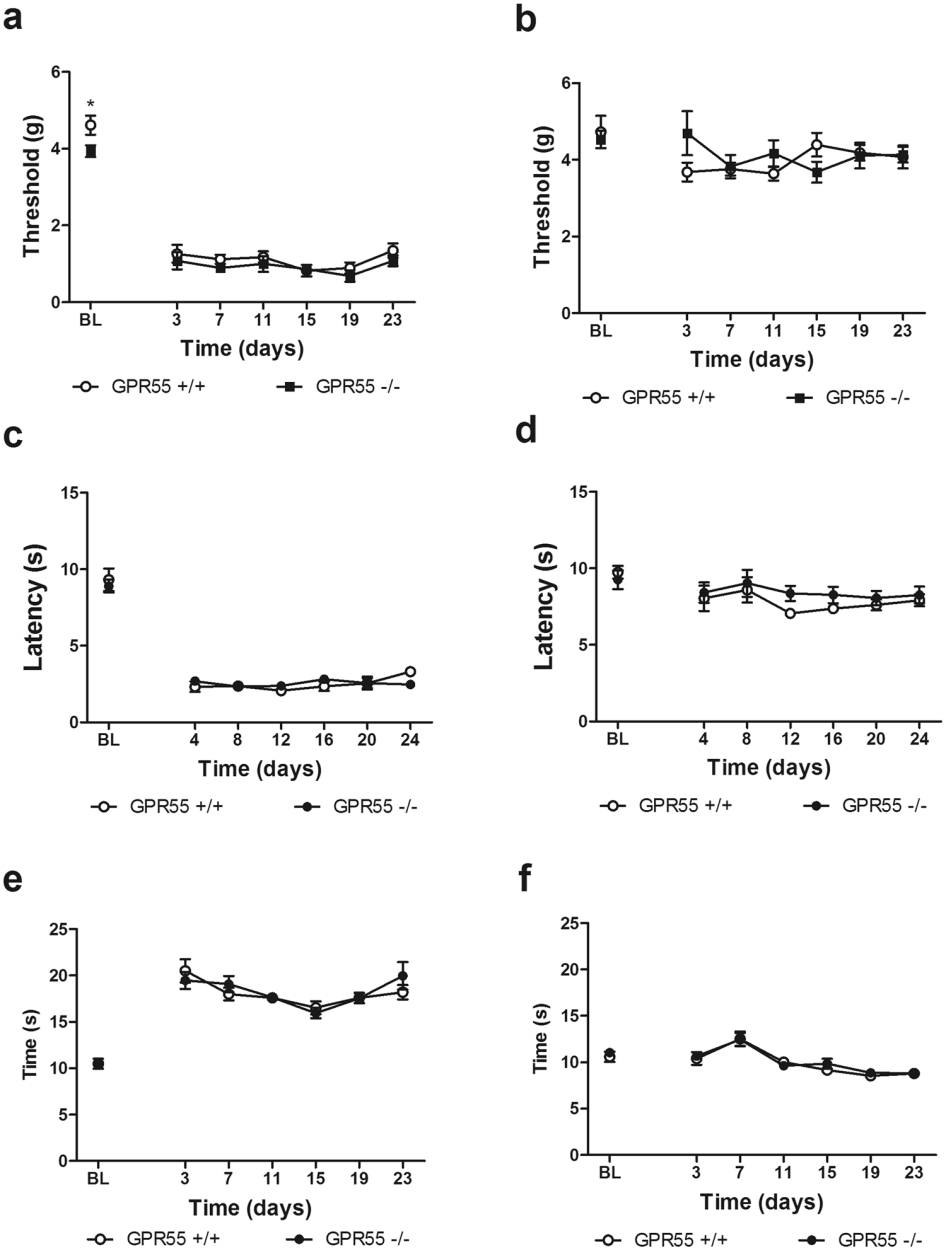
GPR55 KO and WT mice develop similar levels of hypersensitivity to mechanical, heat, and cold stimulation following a partial sciatic nerve ligation. GPR55 KO and WT mice develop similar levels of mechanical (**a**), heat (**c**) and cold allodynia (**e**) in the paw ipsilateral to PSNL. Responses of the contralateral paw to mechanical (**b**), heat (**d**) and cold (**f**) stimulation did not differ between genotypes. Data are expressed as mean ± SEM (n = 7–8 per group). 2-way ANOVA. 2-tailed t-test. *p < 0.05 vs. GPR55 KO.

GPR55 KO and WT mice develop mechanical hypersensitivity in response to PSNL (F_6,78_ = 102.4, p < 0.0001; Fig. 7a). Two way ANOVA revealed that mechanical paw withdrawal thresholds differed across time relative to baseline (F_1,78_ = 8.856, p < 0.05; Fig. 7a) but the interaction between genotype and time post traumatic nerve injury was not significant (p > 0.7; Fig. 7b). GPR55 KO mice displayed a modest reduction in mechanical paw withdrawal thresholds relative to WT mice (t_13_ = 2.413, p < 0.05; Fig. 7a) prior to PSNL but did not differ from WT mice at any time point following PSNL. Traumatic nerve injury did not alter mechanical paw withdrawal thresholds in the paw contralateral to nerve ligation (p > 0.1; Fig. 7b), and there was no effect of genotype (p > 0.6; Fig. 7b) or interaction (p > 0.1; Fig. 7b).

GPR55 KO and WT mice develop hypersensitivity to heat in response to PSNL (F_6,78 = _106.1, p < 0.0001; Fig. 7c). Levels of heat hypersensitivity did not differ between GPR55 KO and WT mice (p > 0.9; Fig. 7c) and the interaction was not significant (p > 0.4; Fig. 7c). In the paw contralateral to PSNL, PSNL altered paw withdrawal latencies over time (F_6,78_ = 2.922, p < 0.05; Fig. 7d), but there was no significant effect of genotype (p > 0.2; Fig. 7d) or interaction (p > 0.7; Fig. 7d).

GPR55 KO and WT mice develop hypersensitivity to cold stimulation in response to PSNL (F_6,78_ = 39.22, p < 0.0001; Fig. 7e). However, genotype did not affect cold hypersensitivity (p > 0.7; Fig. 7e), and there was no significant interaction between time post PSNL and genotype (p > 0.4; Fig. 7e). PSNL also altered sensitivity to cold in the paw contralateral to PSNL (F_6,78_ = 14.19; Fig. 7f). However, genotype did not affect cold responsiveness (p > 0.4; Fig. 7f), and there was no significant interaction between time post PSNL and genotype (p > 0.9; Fig. 7f).

## Discussion

In a previous report, GPR55 KO mice failed to develop mechanical hyperalgesia following either CFA-induced inflammation of the hind paw or PSNL using responding in the Randall-Selitto test as the dependent measure^8^. By contrast, the present studies demonstrate, that in comparison to WT mice, GPR55 KO mice develop and maintain equivalent levels of hypersensitivity to a range of stimuli (i.e. assessed using heat, cold and mechanical stimulation) in multiple mouse models of inflammatory and neuropathic pain using an extensive battery of tests. Overall, hypersensitivity developed in GPR55 KO mice at levels equivalent to those observed in WT mice in every test performed with the exception of the hotplate test. Similar to previous reports^8^, female GPR55 KO mice displayed decreases in thermal response latencies in the hotplate (at 50 and 55 °C) assay, suggestive of hypersensitivity (and/or a facilitated locomotor response) to heat in female mice only. In the present study, GPR55 KO mice displayed normal development of mechanical and heat hypersensitivity following intraplantar injection of capsaicin. In fact, rather than observing an analgesic phenotype (i.e. attenuation of capsaicin-evoked nocifensive behavior), we observed a trend towards increased acute nocifensive behavior in GPR55 KO relative to WT mice. GPR55 KO mice likewise displayed equivalent levels of formalin-evoked pain as well as CFA-induced mechanical allodynia and thermal (heat) hyperalgesia that were comparable to those observed in WT littermates. GPR55 KO mice also displayed levels of paclitaxel-induced mechanical and cold allodynia that were comparable to that observed in WT counterparts. As previous reports only examined responsiveness to mechanical stimulation in the Randall-Selitto test in CFA and PSNL models^8^, another goal of the present study was to test whether the reported deficit was specific for mechanical sensitivity or the pain model assessed. This objective was achieved by measuring the development of hypersensitivity to heat, cold and mechanical stimulation in GPR55 KO mice using multiple models of neuropathic and inflammatory pain. However, we found that GPR55 KO and WT mice did not differ in the development or maintenance of CFA-induced mechanical allodynia following unilateral hind paw injection of CFA. Paw diameters (measured to assess paw edema) were also similar between genotypes in the injected paw. In an independent replication performed by a separate, trained investigator, GPR55 KO and WT mice also exhibited similar levels of CFA-evoked thermal and mechanical hypersensitivity. Thus, the same conclusions were derived from testing GPR55 KO and age-matched WT mice in separate cohorts that were 15 and 28 weeks of age. Similarly, GPR55 KO and WT mice developed similar levels of mechanical, heat, and cold hypersensitivity following PSNL.

The results of the present study stand in stark contrast to the only other published report on the development of inflammatory and neuropathic pain states in GPR55 KO mice, which reported a failure of GPR55 KO mice to develop hyperalgesia. The GPR55 KO mouse used here was a different KO mouse than that used by Staton and colleagues^8^. The GPR55 KO mouse used by Staton and colleagues was generated by deleting amino acids corresponding to the majority of the transmembrane domains of the receptor, while the GPR55 KO mouse used in the present study was generated by deleting exon 2 of the GPR55 gene (the entire coding region for the receptor)^22^. Because experimenters in our study were blinded to genotype and evaluated sex and age-matched GPR55 KO and WT mice in multiple neuropathic (PSNL, paclitaxel-induced neuropathic pain) and inflammatory (formalin, capsaicin, CFA) pain states with multiple dependent measures (mechanical, cold and heat responsiveness) it is unlikely that obvious experimental confounds contribute to the pattern of results obtained here (i.e. preserved nociceptive responding in inflammatory and neuropathic pain states). Nonetheless, it is important to emphasize that, methodological differences do exist between the current study and this previous report. In measuring mechanical hyperalgesia, Staton *et al*. utilized the Randall-Selitto method^23^, which requires that mice are manually restrained to conduct the test, while the present study used an electro von Frey anesthesiometer to measure the threshold for paw withdrawal to punctate mechanical stimulation applied to the plantar paw surface in mice that were not manually restrained. Manual restraint could alter endogenous analgesic tone in the study by Staton and colleagues. In the Randall-Selitto method, an animal is typically restrained, and the paw is pinched between two platforms. By contrast, in the present study, mechanical stimulation was applied to the plantar paw surface via a small diameter plastic tip of an electro von Frey anesthesiometer; mice were able to move freely in the test apparatus to remove the paw and terminate the stimulation. This experimental difference could contribute to the lack of withdrawal response in GPR55 KO mice reported by Staton and colleagues^8^. More work is necessary to determine if GPR55 KO mice might exhibit enhanced stress-induced analgesia^24, 25^.

Genetic deletion of GPR55 has previously been reported to cause problems in motor coordination^10^, with GPR55 KO mice displaying modest impairments in motor coordination in the rotarod test and in the parallel rod footslip test^10^. In the present study, using the same dependent measure assessed by Staton and colleagues (best performance time in the rotarod test), we detected both genotype and sex differences in rotarod performance, and the interaction approached significance (p = 0.06). Male mice overall performed worse than female littermates in the rotarod assay irrespective of genotype, a result that may be due, in part, to the increased body weight in male mice. The observation of increased body weight in male versus female mice (irrespective of genotype) was also reported by Wu and colleagues^10^. Given the modest motor deficits previously observed in GPR55 KO mice (which presented rotarod data from male and female mice pooled across sexes for each genotype)^10^, it is also possible the differences between methods used to deliver mechanical stimulation could contribute to differences between studies (i.e. both to the failure of GPR55 KO mice to develop mechanical hyperalgesia in the previous report^8^ and our observation of preserved mechanical hypersensitivity in our models of inflammatory (capsaicin, CFA) and neuropathic pain (PSNL, paclitaxel-induced neuropathic pain) in the present study). It is reasonable to conceive that the paw withdrawal response for a restrained animal with the limb pinched between two foreign objects being applied with force might be more difficult to execute than a freely moving animal that needs only to lift the paw slightly to terminate stimulation. It is also important to note that differences in rotarod performance were not detected in the previous study by Staton and colleagues^8^, but were detected in studies by Wu and colleagues that used the same GPR55 KO mouse used here^10^. In the present study, the failure to observe robust alterations in motor behavior as a function of genotype as observed by Wu and colleagues could be due to differences in analyses and/or methodology. While Wu *et al*. analyzed performance over four trials given on two separate days^10^, pooling data from male and female mice within each genotype, the present study conformed to the methods reported by Staton *et al*.^8^ wherein mice received three trials on one day, and the longest latency to descent for each mouse was used for analyses. Differences in body weight could possibly account for the effect of sex on rotarod performance observed in the current study. Male mice in the present study weighed significantly more than female littermates, which could have made rotarod performance more difficult. Additionally, performance improved across the three trials performed in the current study (data not shown). Given additional trials, performance may therefore have improved to the levels observed by Wu and colleagues.

Elucidating the precise physiological functions of GPR55 remains problematic^9^ due in part to the lack of unambiguously selective pharmacological tools to probe its function in the intact nervous system. Local injection of a putative GPR55 inverse agonist CID16020046 in the anterior cingulate cortex (ACC) of rats produced antinociception and reduced extracellular signal-regulated kinase 1/2 (ERK 1/2) phosphorylation in the ACC and spinal c-Fos mRNA expression^26^, although mediation by GPR55 was not confirmed using target-specific siRNA knockdown or KO controls. The putative GPR55 agonist O-1602 has been reported to produce anti- or pro-inflammatory effects depending on the model tested. In one report, O-1602 did not alter basal mechanical withdrawal thresholds but blunted the antinociceptive effects of ethanol in a model of neuropathic pain induced by a chronic constriction injury^27^. However, in this latter study^27^ the actions of O-1602 were not shown to be mediated by GPR55. Conversely, O-1602 has also demonstrated anti-inflammatory effects. In a rat model of acute arthritis, O-1602 reduced movement-evoked firing of C fibers in a manner that was insensitive to CB1 and CB2 blockade, but was reportedly reduced by the putative GPR55 antagonist O-1918^28^. Similarly, O-1602 had protective effects in a murine model of experimentally induced colitis^29^. However, these anti-inflammatory effects were preserved in GPR55 KO mice, indicating the anti- and pro-inflammatory effects of O-1602 may be mediated through targets other than GPR55. Likewise, lysophosphatidylinositol (LPI), one putative endogenous ligand for the GPR55 receptor^30^, may also produce off target effects that may confound evidence for the role of GPR55 in pain modulation. Support for the pro-nociceptive role of LPI comes from evidence that application of LPI produces depolarization of periaqueductal gray (PAG) neurons in midbrain slice preparations and reduces nociceptive latencies in the hot plate assay when administered directly into the PAG^31^. Similarly, LPI also increases excitability in peripheral sensory nerves and produces hypersensitivity to mechanical stimulation *in vivo* without eliciting spontaneous nocifensive behavior when administered i.pl.^32^. However, GPR55 KO mice still develop LPI-induced allodynia in a manner dependent on dose of LPI and force of mechanical stimulation, whereas deletion of Gα_q/11_ or Gα_13_ from sensory neurons has a greater effect on removing LPI-evoked tactile allodynia^32^. Thus, GPCR’s other than GPR55 are likely to be involved in the generation of LPI-evoked allodynia. LPI has also been demonstrated to signal through GPR119^33^ as well as non-GPRC targets such as TRAAK, TREK-1^34^, and the Na^+^-Ca^2+^ exchanger^35^ all of which are implicated in nociceptive processing^36–38^. The development of potent and selective GPR55 ligands may reveal the exact function of this receptor in regulating nociceptive processes, but pharmacological evidence gathered so far into the role of GPR55 in nociceptive processes should be interpreted with caution.

GPR55 activation influences ERK 1/2^30^, and intracellular Ca^+2^ signaling^11^, as well as the activity of the small GTPases RhoA^11^ cdc42 and Rac1^3^. As these signaling mechanisms are critical for proper CNS development^39^, GPR55 is positioned to play an important role in neural development. GPR55 has been demonstrated to modulate the growth rate of retinal ganglion cells, and their navigation towards innervation targets^40^. Critically for the context of the current study, GPR55 mediates the guidance of sensory neurons innervating spinal dorsal horn circuitry involved in nociceptive processing, although the impact on pain behavior was not assessed in these studies^41^. Guy and colleagues identified the hydrophilic glycerophospholipid, *lyso-*phosphatidyl-β-D-glucoside (LysoPtdGlc), as a GPR55 ligand released from spinal radial glia that guides appropriate spatial distribution of nociceptive vs. proprioceptive axonal sprouting. In the absence of GPR55 or LysoPtdGlc, nociceptive neurons migrated away from appropriate sites in the dorsal horn, extending dorsomedially into regions typically occupied by proprioceptive afferents. Erroneous wiring of nociceptive circuitry could potentially account for the lack of hyperalgesia previously observed in GPR55 KO mice^8^, but would not explain why responses to mechanical, cold and heat stimulation were intact in our models of inflammatory and neuropathic pain and why no changes in formalin or capsaicin-evoked nocifensive behaviors were observed. Mice used in the current study tended to be older than those used in the previous report (~10 weeks)^8^. Although KO and WT mice were largely age matched within each study, the range of mouse ages evaluated here spanned from 8 weeks to 28 weeks. Nonetheless, separate studies evaluating GPR55 KO mice in the CFA model at 15 and 28 weeks of age produced identical results. More work is necessary to determine whether nociceptive responding changes over the lifespan.

In conclusion, inflammatory and neuropathic pain behavior was preserved in adult GPR55 KO mice relative to WT mice. The same conclusion was derived from our assessment of responses to diverse inflammatory agents (i.e. formalin, capsaicin, CFA) and multiple models of neuropathic pain (i.e. paclitaxel and traumatic nerve injury). The same conclusion was also derived from our assessments of inflammatory and neuropathic pain in GPR55 KO and WT mice that employed multiple modalities of cutaneous stimulation (i.e. mechanical, cold and heat hypersensitivity). The same conclusion was also obtained in mechanistically distinct models of neuropathic pain that either develop or fail to develop hypersensitivity to heat (i.e. PSNL is marked by hypersensitivity to mechanical, cold and heat stimulation whereas paclitaxel-induced neuropathic pain is marked by hypersensitivity to mechanical and cold but not heat stimulation). The development of GPR55 selective ligands, and future research into how genetic deletion, or pharmacological activation of GPR55 alters nociceptive circuitry could bring to focus the nature of this receptors relationship to pathological pain. Our studies using GPR55 KO mice do not implicate GPR55 as a broad-spectrum analgesic target. Further studies incorporating selective pharmacological tools and/or conditional knockout mice will be necessary to fully probe the function of GPR55 in adult animals.

## Methods

### Subjects

One hundred and fifty adult GPR55 KO and WT mice, of both sexes, were used in these experiments. GPR55 KO mice obtained from TIGM (Texas A&M Institute for Genomic Medicine) were generated as described previously^10, 22^. GRP55 KO and WT mice were bred and genotyped at Indiana University. Mice used for the experiments were backcrossed onto the C57BL/6 strain for at least 20 generations. GPR55 KO and WT littermates were used in the same experiments whenever possible. Additional age-matched adult C57BL/6 J mice were purchased from Jackson Labs (Bar Harbor, ME) to supplement results in WT mice. All procedures were approved by the Indiana University Bloomington University Animal Care and Use Committee and followed the guidelines of the International Association for the Study of Pain^42^. Behavioral testing was performed by an investigator blinded to genotype. A single experimenter collected the data shown in each figure panel represented in this report.

### Drugs and chemicals

Capsaicin, formalin, acetone, and Freund’s complete adjuvant (CFA) complete cell suspension were purchased from Sigma Aldrich (St. Louis, MO). Paclitaxel was purchased from Tecoland (Irvine, CA). Capsaicin (1 µg) was dissolved in vehicle consisting of 7% Tween 80 in saline, sonicated, then filtered through a 0.2 µm Millipore filter and administered in a volume of 10 µl. Formalin (37% formaldehyde in water) was diluted directly from stock to 2% in sterile saline and administered in a volume of 10 µl. CFA was diluted 1:1 in sterile saline and administered in a volume of 20 µl. Paclitaxel was dissolved in a vehicle consisting of cremophor EL (Sigma-Aldrich, St. Louis, MO), 95% ethanol (Sigma Aldrich, St. Louis, MO) and saline at a ratio of 1:1:18 respectively in a volume of 5 ml/kg.

### Rotarod test

Motor performance was assessed using an accelerating Rotarod (IITC Life Sciences) (4–40 RPM, 300 second cutoff time). Male (n = 9–10 per group) and female (n = 13–15 per group) mice were placed on the rotarod and the latency to fall was recorded. Mice were given three trials on one day and the best latency to descent is reported to conform to methods published previously by Staton and colleagues^8^.

### Hot plate

Baseline thermal nociceptive latencies were determined using the hot-plate test in male (n = 9) and female WT (n = 15) mice, and male (n = 10) and female (n = 13) GRP55 KO mice. Mice were confined within a clear Plexiglas observation chamber on an IITC hot/cold plate analgesia meter (Woodland Hills, CA), and the latency to the onset of pain behaviors (paw licking, lifting, shaking, tapping, escape behaviors) was measured. Trials were terminated upon observation of pain behaviors or once 40 seconds (60 s for 50 °C) had elapsed. Measurements were taken at 50, 52.5, and 55 °C in the same mice with 24–48 hours separating each testing session. As female GPR55 KO mice displayed baseline differences in thermal nociceptive latencies in this assay, subsequent experiments using heat as a stimulus in pathological pain models were conducted using male GPR55 KO mice only.

### Hargreaves test of plantar heat sensitivity

Paw withdrawal latencies to radiant heat were assessed using the Hargreaves test^43^. Mice were placed in clear Plexiglas cages on an elevated glass platform and allowed to habituate for at least 20 minutes to the chamber prior to testing. A beam of infrared radiant heat (thermal intensity: 28) was applied was applied to the midplantar region of the hind paws using a commercial apparatus (Ugo Basile, Varese, Italy). Stimulation was terminated upon withdrawal of the paw, or after 20 seconds had elapsed. Baseline thermal paw withdrawal latencies were measured in duplicate with 7 minute intervals separating successive stimulations.

### Mechanical paw withdrawal thresholds

Assessment of paw withdrawal thresholds to mechanical stimulation was performed using an electronic von Frey anesthesiometer (IITC model Alemo 2390–5, Woodland Hills, CA) as described in our previously published work^44–46^. This method permits assessment of paw withdrawal thresholds from mechanical stimulation in animals that are not manually restrained. Mice were placed on an elevated mesh table and confined to clear Plexiglas observation chambers. Following at least 20 minutes of habituation to the test chamber, paw withdrawal thresholds were assessed by applying the mechanical stimulator to the midplantar region of the hind paw with a semiflexible tip attached to the anesthesiometer. Stimulation was terminated once the animal withdrew its paw from the stimulus. Paw withdrawal thresholds were measured in duplicate and are reported as the mean of duplicate measurements for each paw averaged across subjects.

### Sensitivity to Cold

Sensitivity to cold was assessed using the acetone method as described previously^44–46^. Cold responsiveness was assessed by applying a drop of acetone to the mid-plantar region of the hind paw through the floor of a mesh platform. The hub of a 1 cc syringe containing acetone with no needle was placed in contact with the plantar surface of the hindpaw. Time spent elevating/licking/biting/shaking the stimulated paw was measured with a stop watch. Each paw was stimulated five times consecutively, allowing 5 min to elapse between successive paw stimulations.

### Capsaicin-evoked mechanical and thermal hypersensitivity

Capsaicin-evoked nocifensive behavior, thermal (heat) hyperalgesia and mechanical allodynia testing were measured as previously described^46, 47^. Because modest but reliable differences in thermal nociception were discovered in the hotplate test in female GPR55 KO mice, male GPR55 KO (n = 6) and male WT (n = 6) mice were used for assessments of sensitivity to heat. Following assessment of baseline responsiveness, mice received a single unilateral intradermal injection of capsaicin (1 µg/10 µL). Starting immediately after capsaicin injection, nocifensive behaviors (lifting, licking, shaking, biting, and tending to the paw) were measured for 5 minutes in the same mice used to assess sensitivity to thermal (heat) stimulation. Paw withdrawal latencies to heat were assessed in duplicate at 10, 30, 45, 60, 90 and 120 minutes post-capsaicin and are reported as the mean of the two duplicate measurements, averaged across subjects.

Our previous work revealed that male and female WT mice of the same C57 background strain did not differ in development of capsaicin-evoked heat or mechanical hypersensitivity^47^. Therefore, female GPR55 KO (n = 6) and WT (n = 6) mice were used to assess possible genotype differences in capsaicin-induced mechanical hypersensitivity. Following assessment of baseline (pre-capsaicin) paw withdrawal thresholds, which did not differ between genotypes, mice received a single unilateral intradermal injection of capsaicin (1 µg/10 µL). Paw withdrawal thresholds were reassessed in duplicate at 10, 30, 45, 60, 90, and 120 minutes post-capsaicin.

### Formalin-evoked tonic pain

Female GPR55 KO (n = 6) and WT mice (n = 6) were placed in a clear Plexiglas chamber on an elevated platform and allowed to habituate for 15 minutes. After habituation, 2% formalin (10 µl) was injected into the superficial surface of the left hind paw. Immediately following formalin injection, nociceptive behaviors were scored for 60 minutes by an investigator blinded to genotype. Composite pain scores (CPS) were calculated for each 5 minute bin using the following criteria: no behavior was scored as 0, lifting of the paw was scored as 1, and biting/shaking/flinching of the paw was scored as 2. The area under the curve of pain behavior was calculated for the early phase (0–10 minutes), and the late phase (10–60 minutes) of formalin pain for each mouse.

### Paw diameter

Paw diameters were measured with a caliper in duplicate and averaged for each paw before and at various time points following CFA administration (i.e. day 1, 2, 3, 4, 7, 11, 12, 15, 16, 19, 20, 23, 24 post injection).

### CFA-induced inflammatory nociception

Following establishment of baseline mechanical paw withdrawal thresholds, mixed sex groups of GPR55 KO (n = 9) and WT (n = 8) mice received a unilateral intraplantar (i.pl.) injection of CFA (20 µl) in the left hind paw. Each 20 µl injection of diluted CFA (1:1 in saline) contained 0.1 mg of *Mycobacterium tuberculosis*. Mechanical paw withdrawal thresholds were reassessed three hours after CFA injection, and on days 4, 8, 12, 16 and 20 following intraplantar injection.

Given that baseline differences in thermal nociception were observed in the hotplate test in female GPR55 KO mice, a separate study was performed to evaluate male GPR55 KO (n = 8), and WT mice (n = 8) for CFA-induced inflammatory nociception to mechanical and heat stimulation by a separate blinded experimenter. CFA-induced thermal and mechanical responsiveness was assessed using the same mice. Following acquisition of baseline mechanical paw withdrawal thresholds and thermal paw withdrawal latencies, mice received a unilateral i.pl. injection of CFA (20 µl). Mechanical paw withdrawal thresholds were measured in duplicate three hours after CFA injection and again on days 3, 7, 11 and 15 following CFA injection. Thermal paw withdrawal latencies were measured in duplicate 3 h after CFA injection and again on days 1, 4, 8, 12, and 16 post-injection.

### Paclitaxel-induced neuropathic pain

To induce chemotherapy-induced neuropathic pain, GPR55 KO mice (n = 8 female) and WT mice (n = 5 (3 female, 2 male)) received systemic administration (i.p.) of paclitaxel (1 mg/kg/day) on four consecutive days. Paw withdrawal thresholds were measured before and on day 2, 4, 6, 8, 12, 16, 20 and 24 following the start of paclitaxel administration^44–46^. Cold responsiveness was assessed using the acetone method in the same mice used to assess mechanical paw withdrawal thresholds as described previously^44–46^. Time spent attending to the acetone-stimulated paw was assessed on days 4, 8, 12, 16, 20 and 24 following the initiation of treatment with either paclitaxel or its cremophor-based vehicle. As a control, a subset of mice (3 male GPR55 KO, 1 female WT, 2 male WTs) also received the cremophor-based vehicle in lieu of paclitaxel.

### Traumatic nerve injury

Following determination of baseline measurements to mechanical, heat or cold stimulation, mice underwent surgical procedures to induce a partial sciatic nerve ligation (PSNL) using the Seltzer model^20^. Mice were deeply anesthetized with 2.5% isoflurane, shaved, and an incision was made at the level of the mid-thigh. Blunt dissection was performed to expose approximately 1 cm of the left sciatic nerve. A suture (10–0) was passed through the dorsal third of the nerve and tightly ligated. Fascia and musculature were closed with sterile silk sutures and the skin was closed with wound clips. The mice were allowed 3 days to recover prior to testing. As female GPR55 KO mice displayed baseline differences in thermal nociceptive latencies, male GPR55 KO (n = 8), and mixed sex WT (n = 7) mice were used to assess PSNL-induced mechanical, cold, and thermal responsiveness. Mechanical paw withdrawal thresholds and duration of cold responsiveness were assessed on days 3, 7, 11, 15, 19 and 23 post-surgery while thermal (heat) paw withdrawal latencies were performed on days 4, 8, 12, 16, 20 and 24 post-surgery.

### Statistical analysis

A 2 × 2 ANOVA was used to assess differences in rotarod descent latencies (i.e. best time) in male and female GPR55 KO and WT mice. Two way repeated measures ANOVA was used to assess differences in rotarod descent latencies across trials. Hot plate responding was assessed in male and female mice separately using two way repeated measures ANOVA. Unpaired T-tests were performed to assess possible differences in capsaicin-evoked nocifensive behavior. Two-way repeated measures ANOVA were used to measure the development of hyperalgesia following capsaicin, formalin, CFA, paclitaxel and PSNL. Statistical analyses and figures were generated using GraphPad Prism version 5 (GraphPad Software Inc., La Jolla, CA, USA). Statistical significance was defined as p < 0.05.
